# EEG channel selection using metaheuristic algorithms in alcoholism detection using optimal wavelet transform

**DOI:** 10.1038/s41598-026-49177-9

**Published:** 2026-05-18

**Authors:** Digambar V. Puri, Pramod Kachare, Sandeep Sangle, Surendra Solanki, Deepak Panwar

**Affiliations:** 1 Department of Information Technology, Vidhyalankar Institute of Technology, Wadala, Mumbai, Maharashtra 400 037 India; 2 Department of Electronics and Telecommunication, Ramrao Adik Institute of Technology, D. Y. Patil University, Navi-Mumbai, Maharashtra 400706 India; 3 Department of Artificial Intelligence and Machine Learning, Manipal University Jaipur, Jaipur, Rajasthan 303007 India

**Keywords:** Alcoholism, Discrete wavelet transform, Electroencephalogram, Filter banks, Halfband filter, Support vector machine, Computational biology and bioinformatics, Engineering, Mathematics and computing, Neuroscience

## Abstract

Alcoholism, habitual and excessive intake of alcoholic beverages, presents a significant disorder that challenges contemporary society; however, alcoholism detection lacks universally acknowledged examinations or protocols. Traditional subjective methods are time-consuming and prone to error. Electroencephalography (EEG) detects alcoholism by analyzing the electrical activity of the brain. An effective EEG channel selection using metaheuristic algorithms (MHAs)-based features are introduced. The EEG signal is decomposed into subbands using a novel optimal wavelet filter bank (OWFB). Each subband is represented using four features: mean, Higuchi’s fractal dimension, log entropy, and Rényi’s entropy. The optimal subband features are investigated using combinations of four MHAs and are six classification models. A publicly available 64-channel EEG dataset of alcoholic and non-alcoholic signals is employed. Among the evaluated combinations, Sparrow search algorithm combined with k-nearest neighbor model (KNN) classifier achieved the highest accuracy of 95.90% and F1-score of 96.80%, closely comparable to using all EEG channels (96.30% accuracy and 96.83% F1-score). Overall, KNN classifier consistently outperformed others, indicating that optimal channel selection can effectively reduce channel redundancy while maintaining high accuracy in alcoholism detection. The optimal channel selection enhances the performance of the ML models and reduces the computational time compared to existing alcoholism detection methods. A Python implementation is available at https://github.com/pramodkachare/EEG_Optimal_Wavelet_MHA.

## Introduction

Alcoholism is a condition caused by an individual’s habitual and excessive intake of alcoholic beverages. Chronic alcohol intake creates dependency in individuals, leading to alcohol addiction and even death^1^. Alcoholism is progressing in stages throughout the community, affecting people of every class of society. According to a World Health Organization study report, roughly 2 billion human beings worldwide consume alcoholic beverages, with 76.3 million of them suffering from alcohol dependency syndrome. Approximately 58.3 million individuals live with a disability, while drinking is responsible for 1.8 million fatalities (total deaths)^2^. Alcoholism can cause diabetes, neurological disorders, pregnancy complications, liver damage, pancreatic disease, and accidental injuries^3^ (Table 1).

**Table 1:** Various important contributions for the detection of alcoholism.

Reference	Method	Features Extracted	Classification Model(s)	Accuracy
Shooshtari et al.^4^	Spectral analysis	MaG power features	SVM	82.98%
Acharya et al.^5^	–	APEN, SAEN, higher order spectrum	SVM	91.70%
Faust et al.^6^	WPT	subband energy features	KNN, GMM, PNN, DT	95.80%
Mumtaz et al.^7^	Spectral analysis	Absolute and relative band power	SVM, LDA, MLP	96.00%
Bajaj et al.^8^	Time-frequency	Statistical features	NNLS	95.83%
Padma Shri et al.^9^	FFT	Spectral entropy	K-NN	95.50%
Taran et al.^10^	EMD rhythms	Statistical features	ELM	97.93%
Priya et al.^11^	EMD	Statistical features	Least square SVM	97.92%
Bavkar et al.^12^	Spectral analysis	Absolute Gamma band power	Ensemble KNN	95.10%
Bavkar et al.^13^	EMD	Bandwidth features with DBHS	Ensemble subspace K-NN	93.87%
Malar et al.^14^	Wavelet transform	Statistical features	MLP, RBFN, ELM	87.60%
Sharma et al.^15^	DT-CWT	Various subbands-based features	SVM, LS-SVM, ELM	97.91%
Sangle et al.^16^	Power band XAI	Power subbands	ANN+XAI	97.36%

*APEN* approximate entropy, *SAEN* sample entropy, *GMM* Gaussian mixture model, *SVM* support vector machine, *RBFN* radial basis function, *LS-SVM* least square SVM, *EMD* empirical mode decomposition, *DT-CWT* dual tree continuous wavelet transform, *WPT* wavelet packet transform, *GMM* Gaussian mixture model.

Prolonged alcohol consumption negatively affects the maturation of the human brain over time^17^. In the short term, alcohol use leads to various problems, such as memory lapses, temporary cognitive blackouts, increased impulsiveness, and compromised judgment. The National Institute on alcohol abuse and alcoholism states that consistent alcohol abuse results in reduced cognitive function, impaired visuospatial skills, and the onset of memory degradation^18^. Identifying alcoholism proves intricate, and a universally acknowledged protocol for its detection remains absent. While some conventional devices employ olfactory cues, this method often yields inaccurate results. There is no standardized and effectively trustworthy technique for detecting alcoholism in individuals^19^. Diagnosis typically involves a clinical symptoms assessment and a series of tests administered by skilled and experienced medical professionals. However, these traditional methods are time-intensive and subject to human error. In practice, numerous neuroimaging methods can show the ability to detect alcoholism, such as functional magnetic resonance Imaging and Positron emission tomography. However, these methods require more trials and exposure to radiation^20^.

Recent medical practices have extended the application of Electroencephalography (EEG) to detect alcoholism in patients, as discernible differences emerge in brain activity patterns between alcoholic and control subjects. EEG is an effective technique with high temporal resolution and a cost-effective solution to detect alcoholism. EEG employs various electrodes (channels) comprising small metallic discs and wires to gauge cerebral electrical activity. Placed at various positions on the scalp, these electrodes capture voltage fluctuations originating from neuronal activity within the brain. Widely used for studying brain function, EEG serves as a diagnostic tool for various brain conditions and disorders. However, interpreting EEG signals is a complex task. Proficient medical practitioners well-versed in this domain are required to decipher these signals, identifying pertinent patterns that aid in detecting alcoholism.

Overall, the EEG technique scrutinizes electrical activity across diverse scalp sites, generating substantial volumes of multi-channel data^21,22^. Nevertheless, this data is susceptible to noise. Large-scale efforts have been directed towards enhancing the accuracy and reliability of EEG-based alcoholism detection techniques. The substantial presence of noisy data amplifies the intricacy of manual classification, rendering the task time-intensive and particularly challenging for proficient practitioners. Another notable drawback of manually scrutinizing EEG signals is their susceptibility to errors. There are instances when even well-trained experts could overlook crucial patterns due to noise interference. Consequently, an imperative arises for an automated framework that can assess and categorize these EEG signals. Such a system should exhibit precision, reduce vulnerability to human error, and necessitate minimal human involvement. In recent years, various state-of-the-art (SOTA) methods have been reported to identify alcoholism from EEG signals. The SOTA methods reported to detect alcoholism have been presented in Table 1. Table 1 shows the feature extraction, channel selection, and obtained results from SOTA methods. Authors have reported various non-linear handcrafted features to detect alcoholism from EEG signals. Acharya et al.^5^ reported the non-linear features with a support vector machine-based alcoholism detection method. Faust et al.^6^ mentioned fast Fourier transform (FFT) based autoregressive (AR) band power features with various machine learning (ML) classifiers to classify the alcoholic and non-alcoholic EEG signals. Bajaj et al.^8^ used short-time FT (STFT) and NNLS (non-negative least square) classifiers to identify the alcoholic subjects. Recently, Cohen et al.^3^ investigated the effect of ensemble learning classifiers with independent component analysis and FFT to identify alcoholism. They performed two different approaches to classify the alcoholic and NC EEG signals using temporal data and derived FFT-based images from the EEG signals. Zhu et al.^23^ reported the visibility graph entropy with a based approach to enhance the alcoholism detection rate. The studies mentioned in^3–23^ utilized features from all the EEG channels to enhance the classification rate of alcoholic and NC subjects. However, this increases the redundant features and computational time. Therefore, to identify the significant EEG channels, Padma et al.^9^ proposed an EEG channel selection method based on t-test statistics. The spectral features from optimal EEG channels obtained 95.50% accuracy with SVM. Meng et al.^24^ investigated an optimal number of channels using principal component analysis (PCA) with power spectral features with neural network classifiers.

Sadiq et al.^25^ proposed that the phase space reconstruction of EEG signals was utilized to explore their nonlinear and chaotic behavior. The study extracted 34 geometrical and dynamical features to represent the EEG complexity and employed a feedforward neural network (FFNN) optimized through Henry Gas Solubility Optimization (HGSO). Akbari et al.^26^ proposed a depression detection framework that utilized geometrical descriptors extracted from the Second-Order Differential Plot (SODP) of EEG signals. The study computed various geometric measures, including angles between consecutive vectors, centroid distances, and triangle areas, to capture nonlinear temporal variations between depressed and healthy individuals. Using Binary Particle Swarm Optimization (BPSO) for feature selection and KNN/SVM classifiers, the model achieved an accuracy of 98.79%, outperforming conventional time–frequency domain features. These studies demonstrate that geometrical EEG features, when integrated with optimization algorithms and neural classifiers, can enhance model interpretability and performance^27^.

Recent progress in deep learning has shown that pre-trained convolutional neural networks (CNNs)—originally trained on large-scale vision datasets. Mukhtar et al.^28^ developed two CNN-based models—baseline CNN and regularized CNN—for the classification of EEG signals. The Baseline model achieved an accuracy of 91.15%, while regularized CNN improved performance to 98.43%. Despite the enhancement, both models exhibited overfitting and underfitting tendencies. Li et al.^29^ introduced a hybrid framework that integrated a Convolutional Neural Network (CNN) with Discrete Wavelet Transform (DWT)^30^ and Bidirectional Long Short-Term Memory (BiLSTM) networks, attaining an average accuracy of 99.32%. However, their method experienced a degree of information loss during transformation. Nevertheless, their approach was constrained by high computational cost and limited model generalization. This work confirms that pre-trained CNNs can effectively capture spatiotemporal EEG structures while maintaining resilience to data scarcity—an important factor in medical and cognitive EEG applications. The integration of such transfer learning approaches provides a foundation for developing efficient EEG-based diagnostic systems, especially when combined with lightweight or disease-specific architectures like EEGNet and SteadyNet.^31,32^ Several studies have enhanced EEG-based cognitive load detection using advanced signal processing and feature optimization techniques, including empirical wavelet transform^33^, lead-wise feature optimization with ensemble classifiers^34^, and techniques that use hand-crafted feature selection^35^, demonstrating improved classification accuracy and robustness. The phase space dynamics approach is introduced to visualize alcoholic EEG signals with graphical features for classifying normal and alcoholic EEG signals^36^.

**Fig. 1:**
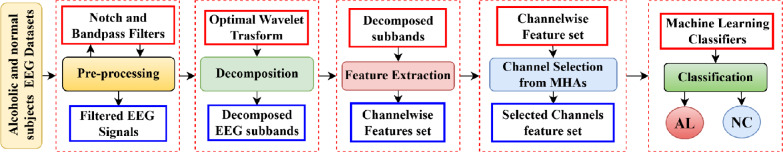
Schematic diagram for the proposed method.

From the above-discussed methods, it is noted that most of the techniques use hand-crafted feature methods and ML models. The selection of these methods and ML models totally depends on the expert knowledge of the researchers. The FFT and STFT-based methods provide poor time-frequency resolution. To address the above issues, the present study proposes an automatic alcoholism detection framework using different metaheuristic algorithms for the EEG channel selection with the optimal wavelet transform. The major contributions of the present technique are listed as follows:

- The NC subjects consisted of 48 males with ages ranging from 20 to 39 years (mean age = 25.81 years), whereas the ages of the alcoholic group ranged from 20 to 50 years using optimal wavelets.
- Enhancing the classification accuracy in alcoholism using highly distinct features compared to SOTA techniques.
- Improving the performance using optimal machine learning classification algorithms.

The rest of the paper is described as follows: Section II includes the details of the methodology and EEG datasets. Section III contains the experimentation and obtained results from the proposed method. The comparative analysis and neurophysiological significance are provided in the discussion section. Finally, the paper has been concluded in the last section.

## Methodology

Figure 1 depicts the suggested automated alcoholism detection architecture. Initially, EEG signals are preprocessed using notch and bandpass filters to remove noise and artifacts. The filtered EEG signals are then decomposed channel-wise using the proposed optimal wavelet filter bank (OWFB) to obtain EEG subbands. The various features are extracted from the decomposed subbands of all the channels to form a channel-wise feature set. The metaheuristic algorithms (MHAs) are then used to select the most informative EEG channels, resulting in a reduced feature set (The details of channel selection has been provided in subsection 2.5). Finally, various ML models were trained and evaluated utilizing the optimum feature set from alcoholic and NC EEG signals. The proposed methods have been tested on the publicly available EEG dataset of alcoholic and normal subjects. The details of this dataset are provided in the subsequent section.

### EEG dataset and pre-processing

**Fig. 2:**
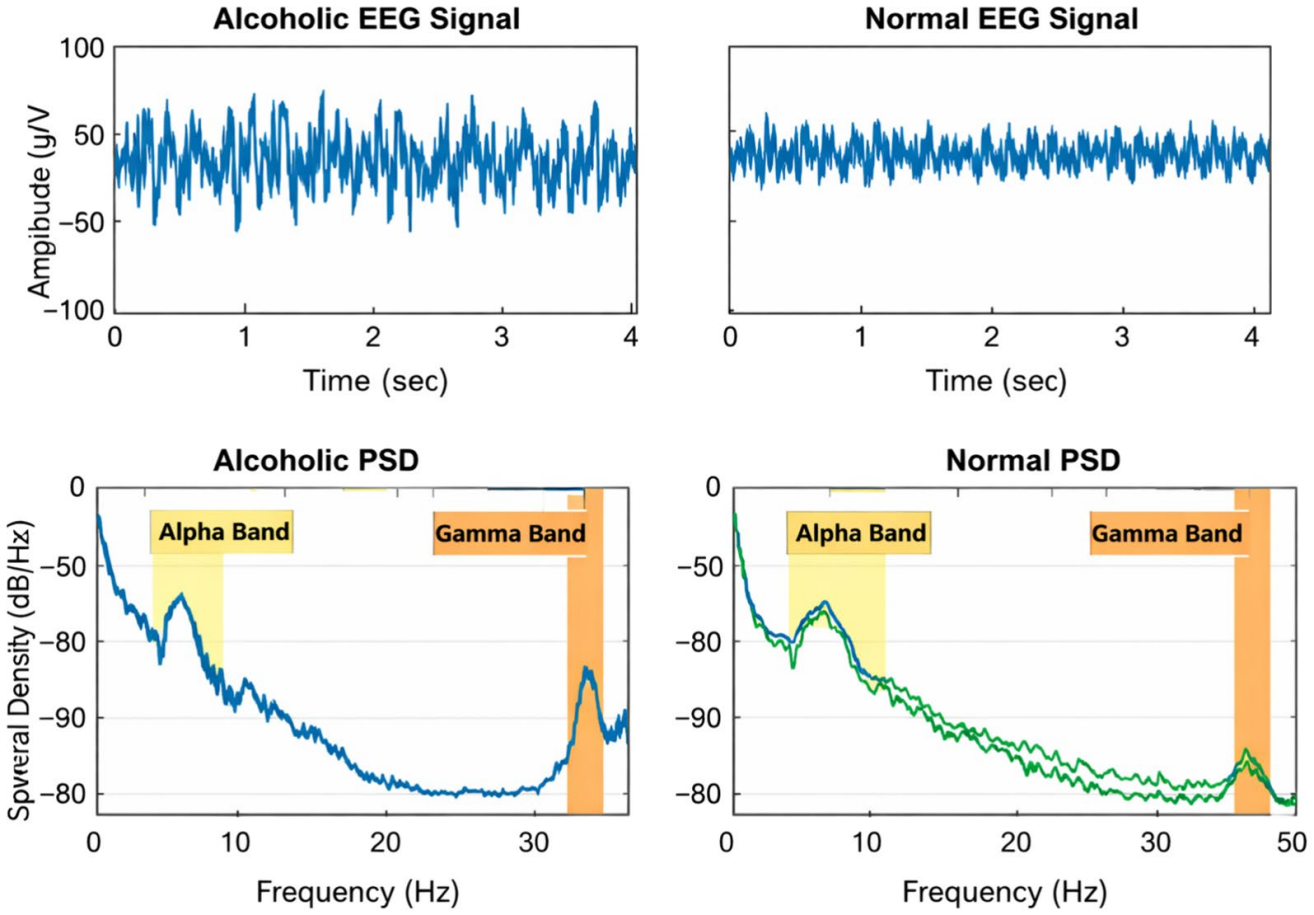
Sample signal of alcoholic and normal EEG recording with spectrum.

The publicly available EEG datasets of alcoholic and normal control subjects used in the present study have been taken from KDD UCI, University of California (https://kdd.ics.uci.edu/databases/eeg/eeg.data.html)^37^. All EEG signals were recorded according to the 10-20 electrode placement system from a total of 122 subjects (77 alcoholic and 45 non-alcoholic) with 120 trials, each comprising 64 channels (electrodes). The NC subjects consisted of 28 males with ages ranging from 20-39 years (mean age = 25.81 years), whereas the ages of the alcoholic group ranged from 20–50 years. Each subject dealt with the standardized set of pictures from the 1980s Vanderwart and Snodgrass set^38^. Each sample length is one second and has a sampling rate of 256Hz. The artifacts, such as eye blinking muscle movements, are rejected using a a fourth-order Butterworth bandpass filter with cutoff frequencies of 0.5–60 Hz (sampling frequency = 256 Hz) was applied for noise removal. Zero-phase filtering was implemented to avoid phase distortion. More details of this dataset are available in^5^. The time domain EEG signals and their power spectral density (PSD) plots are presented in Fig. 2. he PSD plots are presented to demonstrate that these frequency bands are significant and are effectively captured within the wavelet subbands used in the proposed method.

### Optimal wavelets

There are various signal decomposition methods reported in the literature for the analysis and classification of EEG signals. These methods aim to decompose the complex, non-stationary EEG signal into simpler intrinsic components that better represent its temporal and spectral characteristics^39^. Commonly employed approaches include Multivariate Empirical Variational Mode Decomposition (MVMD), and Empirical Wavelet Transform (EWT)^40^, as well as their multivariate extensions. Each technique provides a different trade-off between time–frequency resolution and computational complexity, enabling more effective extraction of task-specific or pathological features from EEG data^41^.

**Fig. 3:**
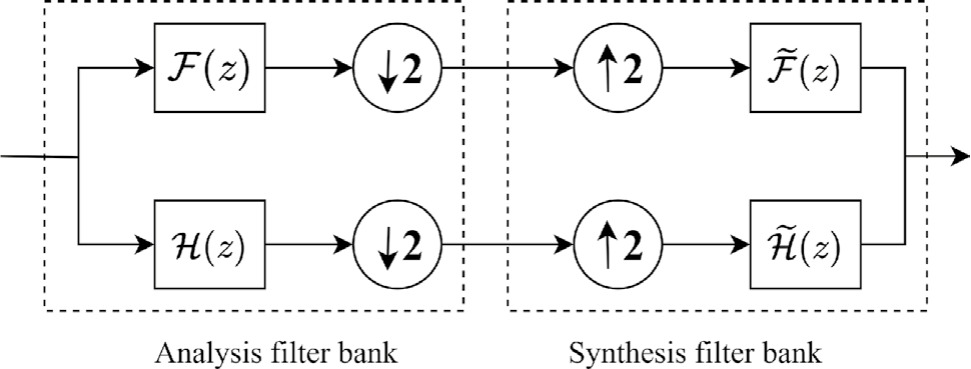
General analysis-synthesis scheme using two-channel filter-bank.

The two-channel filter-bank (FB) is designed using finite impulse response filters, as shown in Fig. 3. Let the $\mathcal {F}(z)$ and $\mathcal {H}(z)$ lowpass filter (LPF) and highpass (HPF) analysis filter, respectively. To have any two-channel FB perfectly reconstructed, one has to follow conditions (2) and (3),

1 $$\begin{aligned} \mathcal {F}(z)\widetilde{\mathcal {F}}(z) + \mathcal {H}(z)\widetilde{\mathcal {H}}(z) = 2z^{-l} \end{aligned}$$

where *l* is a delay.

2 $$\begin{aligned} \mathcal {F}(z)\widetilde{\mathcal {F}}(-z) + \mathcal {H}(z)\widetilde{\mathcal {H}}(-z) =0 \end{aligned}$$

If $\mathcal {F}(z)= \widetilde{\mathcal {H}}(-z)$ and $\widetilde{\mathcal {F}}(z)=-\widetilde{H}(-z)$, then product filter $\mathcal {G}(z) = \mathcal {F}(z)\widetilde{\mathcal {F}}(z)$, therefore (1) will become,

3 $$\begin{aligned} {\mathcal {G}}(z)- \mathcal {G}(-z) = 2z^{-l}\;\;\;\; l = 1,\;3,...\;\;\text {odd} \end{aligned}$$

The filter banks designed for perfect reconstruction have a *delay of odd multiples* (i.e., $l = 1, 3, 5, \dots$). This choice ensures that the signals processed through the filters remain correctly aligned after reconstruction, especially when combining the results of the lowpass and highpass filters.

The OWFBs can be designed using half-band polynomials $\mathcal {G}(z)$. The steps for the design of OWFBs are as follows.

1. Let $\rho$ and $\sigma$ are the lengths of lowpass analysis and synthesis filters, respectively. These lengths should satisfy the criteria $\text {remainder}\{(\sigma +\rho ),4\} =0$.
2. Compute the HBP $\mathcal {G}(z)$ of length $l=\rho +\sigma -1$.
3. compute the unknown $u=(l-1)/2-1$.
4. The maximum vanishing moments of $\mathcal {G}(z)$ can be computed as $m=l-u-1$. This results in $\text {remainder}\{\mathcal {G}(z), (z+1)^m\} =0$.
5. Find the expression for the $Q(z) = (z+1)^m$ and compute vector $\Phi _{1xu}$ containing first *u* coefficients of *Q*(*z*) i.e. $\phi =[1,\;\phi _2\;\phi _3,...,\;\phi _u]$.
6. Evaluate equation,
  4 $$\begin{aligned} \mathcal {T}_{u\times u} \times \mathbb {S}_{u\times 1} = \Psi _{u\times 1} \end{aligned}$$
7. The polynomial can be constructed as:
  5 $$\begin{aligned} \mathcal {D}(z) = s_{\lceil u/2 \rceil } z^{\lceil u/2 \rceil } + \sum _{i=0}^{\lceil u/2 \rceil -1} s_i (z^i + z^{u-i}) \end{aligned}$$
8. Evaluate the roots of $\mathcal {D}(z)$ having phase in $[0, \pi /2]$ range and lie on the unit circle. compute the real $(\mathcal {D}_r)$ and imaginary $(\mathcal {D}_j)$ roots for various values of $\sigma$ and $\rho$. The $\mathcal {D}_r$ and $\mathcal {D}_j$ are given by,
  6 $$\begin{aligned} & \mathcal {D}_r = [\mathcal {D}_{r_1},\;\mathcal {D}_{r_2},\;\;\mathcal {D}_{r_3},...,\;\;\mathcal {D}_{r_k}] \end{aligned}$$
  7 $$\begin{aligned} & \mathcal {D}_j = [\mathcal {D}_{j_1},\;\mathcal {D}_{j_2},\;\;\mathcal {D}_{j_3},...,\;\;\mathcal {D}_{j_n}] \end{aligned}$$
  where *k* and *n* satisfy the relation $2k+n=u/2$.

The possible HBP with different lengths and vanishing moments are presented in 2.

**Table 2:** HBP for various lengths.

m	($\sigma +\delta$)	$\mathcal {D}(z)$
4	8	{1, -4, 1}
6	12	{1, -6, 12.67, -6,1}
8	16	{1, -8, 26.20, -41.6, 26.20, -8, 1}
10	20	{1, -10, 43.5, -104.3, 143.4, -104.3, 43.5, -10, 1}

Once the HBPs are computed, OWFBs with two HBPs can be designed. The creation of wavelets is that both the decomposition and reconstruction LPFs possess at least two zeros at $z =-1$. Additionally, we ensure that the filters are built using real coefficients. The residual roots of the HBP can be utilized in the design of the analysis LPF. This process is then iterated for every feasible combination present. The constructed wavelets are referred to as OWFB($\sigma +\rho ,t$), where *t* is an index of combination. It forms 45 different combinations for $l=15.$ Some of the combinations are presented in Table 3. Table 2 provides the LPF coefficients for few OWFB($\sigma +\rho$, *t*) combinations. It is noted from Table 2 that all coefficients are dyadic; therefore, these are implemented on the hardware with *shifts* and *adds* (Table 4).

**Table 3:** OWFB($\sigma +\rho$, *t*) combinations for $l=15$.

OWFB	*t*	${I}_{re}$	${\tilde{I}}_{re}$	${I}_{im}$	${\tilde{I}}_{im}$	*Z*	$\mathfrak {P}$	Type
	22	1	1	0	0	4	6	B
	32	1	1	0	0	3	5	A
(16, *t*)	36	1	0	0	0	4	5	C
	39	0	0	0	1	2	4	D
	44	0	1	0	0	2	3	C
	47	0	0	0	0	2	2	B
	11	0	0	1	1	4	8	D
	25	0	0	0	1	4	6	D
(20, *t*)	31	0	0	0	1	2	4	D
	32	0	0	1	0	2	4	D
	34	0	0	0	0	3	3	A
	35	0	0	0	0	2	2	B

Type A: even-length symmetric bi-orthogonal wavelets.

Type B: odd-length symmetric bi-orthogonal wavelets.

Type C: even-length asymmetric bi-orthogonal wavelets.

Type D: odd-length asymmetric bi-orthogonal wavelets.

**Table 4:** Different Filter banks with their coefficients designed from the proposed method.

OWFB($\sigma +\rho$, *t*)	Synthesis LPF coefficients
OWFB(16, 22)	$\{-264, -166, 1712, 3229, 1712, -166, -264\}/4096$
OWFB(20, 11)	$\{160,-332, -1103, 1239, 3954, 1971, -338, 17, 223\}/4096$

**Table 5:** The properties comparison of the proposed 9/7 and existing filter banks.

Wavelets	$\sigma /\rho$	$\Delta W$	Adds	Shifts	$E_r$	$\Delta t^2$	$\Delta \omega ^2$	$\Delta t^2$ $\Delta \omega ^2$
DBT^42^	9/7	2.8472	42	58	31.83	0.4202	0.9725	0.3825
CLZ^43^	11/9	2.8365	35	156	35.74	0.4135	0.9962	0.4017
OWFB(16, 22)	9/7	1.4120	65	56	27.45	0.3975	0.9558	0.3565
OWFB(20, 11)	11/9	1.2556	29	38	19.15	0.3846	0.9756	0.3648

#### Computational complexity and property measures

The wavelet filter properties like energy of error ($E_r$), time ($\Delta t^2$), frequency ($\Delta \omega ^2$), and time-frequency ($\Delta t^2\Delta \omega ^2$) localization, transition-width ($\Delta W$) of the OWFBs and earlier existing methods reported by Chang et al. (CLZ)^44^), Tay et al. (DBT^45^), Liu and Ngan (L&N)^46^, and Murugesan et al. (M&T)^47^ are presented in Table 5. OWFBs require lower ‘shifts’ and ‘adds’ to design the same filter bank (FB) (9/7 or 11/9) as existing FBs. This reduces the computational complexity of the filters, resulting in a reduction of computational time. From the Table 5, it is clear that OWFBs exhibit enhanced frequency selectivity, low $\Delta W$, and optimal $\Delta t^2\Delta \omega ^2$ than earlier FBs. Notably, orthogonal wavelets maintain consistent energy distribution across average subbands. Conversely, the efficacy of bi-orthogonal wavelets depends on the length of both decomposition and reconstruction filters.

### Feature extraction

Feature Extraction is a significant step in any classification problem. This process helps to achieve substantial information present in various datasets. In the present study, four features, namely, mean, Higuchi’s fractal dimension (HFD), log entropy, and Renyi’s entropy, have been extracted from EEG subbands^48^.

### Metaheuristic algorithms

**Fig. 4:**
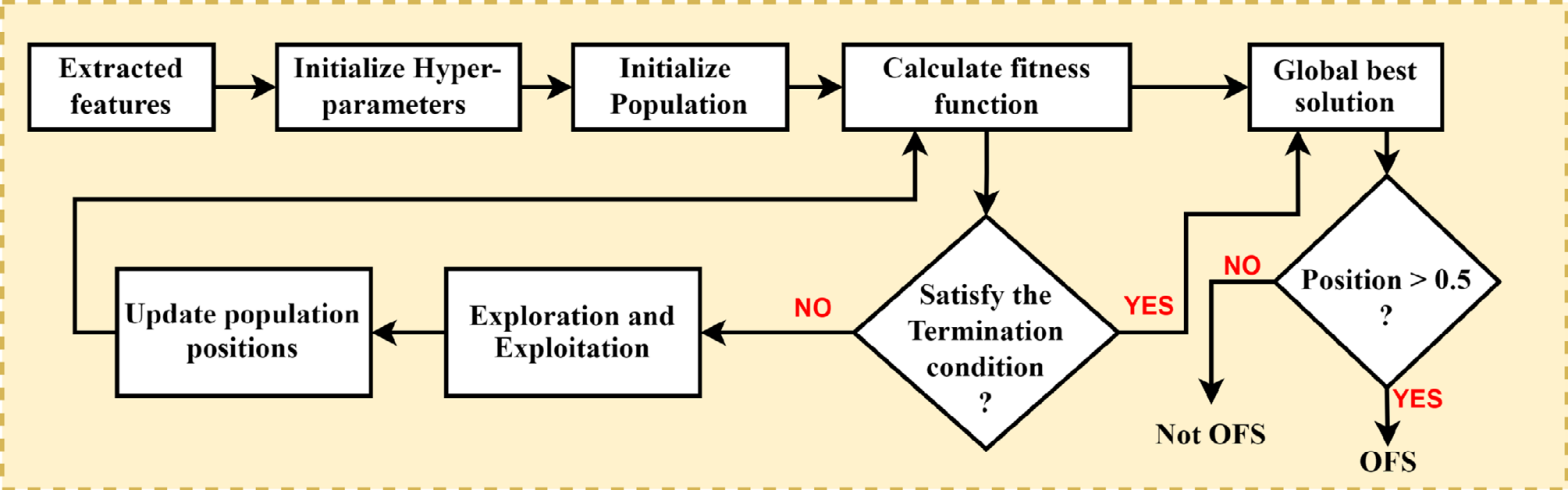
General structure of MHAs.

The general flow of MHAs like RSA, SSA, PSO, and SO is presented in Fig. 4. The details of these MHAs are provided below.

#### Particle swarm optimization (PSO)

PSO is a nature-inspired optimization algorithm that was developed to simulate the social behavior of birds flocking or fish schooling. It falls under the category of swarm intelligence algorithms and is commonly used to solve optimization and search problems. PSO is based on the concept of a group of particles (potential solutions) that move through a multidimensional search space to find the optimal solution. Each particle’s movement is influenced by its own historical best position (personal best) and the best position found by the entire swarm (global best)^49^. Here is a general outline of how the PSO algorithm works:

- Initialization: initialize a population of particles, each with a random position and velocity within the search space. Evaluate the fitness of each particle’s position.
- Main loop: for each iteration (generation), update each particle’s velocity and position based on its personal best and the global best. Update the personal best for each particle if the current position is better than the previous personal best. Update the global best position based on the best fitness value found by any particle in the swarm.
- Update Velocity and Position: The new velocity of each particle is determined by combining its current velocity, personal best position, and global best position. The velocity is adjusted using acceleration coefficients and random values. The new position of each particle is calculated by adding its current position to the updated velocity.
- Termination: the algorithm terminates when a predefined number of iterations is reached or when a certain convergence criterion is met^49^.

#### Reptile search algorithm (RSA)

Reptile is a meta-learning algorithm that aims to improve the performance of machine-learning models on new tasks with limited data. The Reptile algorithm is designed to learn a model that can quickly adapt to new tasks through a process called “inner loop” and “outer loop” optimization. Here is how the algorithm works^50^:

- Initialization: start with an initial model that will be optimized for fast adaptation.
- Inner loop: for each task, sample a small batch of training data. Update the model’s parameters using gradient descent on this task’s data, but only for a few steps (referred to as the “inner loop iterations”). This encourages the model to adapt to the task’s specific characteristics quickly. Save the updated model parameters after a few iterations.
- Outer loop: after updating the model on multiple tasks through the inner loop, compute the average of the saved model parameters. This average represents the meta-learned model that has captured common features across tasks. Update the initial model’s parameters toward this average model to improve its ability to adapt quickly to new tasks. This is done using gradient descent or a similar optimization method^50^.
- Repeat: the inner and outer loop steps are repeated for a certain number of iterations or until convergence^50^.

#### Sparrow search algorithm (SSA)

SSA is a metaheuristic algorithm inspired by the collective behavior of salps, a marine organism. The algorithm mimics the movement and interaction of salps in order to solve optimization problems. In SSA, a population of virtual salps is initialized randomly in the search space, where each salp represents a potential solution to the optimization problem. Salps move in the search space using a combination of exploration and exploitation strategies. The population is categorized as leaders and followers to construct a mathematical model of salp chains. The details of SSA are available in^51^.

#### Snake optimizer (SO)

An innovative metaheuristics algorithm called the Snake Optimizer (SO) has been introduced to address a wide range of optimization challenges by simulating the unique mating behaviors of snakes^52^. In this algorithm, each snake, whether male or female, competes to find the most suitable partner when there is a sufficient food supply and low temperatures. This research employs mathematical modeling to replicate and analyze these foraging and reproduction behaviors, ultimately presenting a straightforward and effective optimization algorithm^52^. The channel section algorithm has been discussed in the subsequent section.

### Channel selection

In the literature, various optimization techniques are used for the features selections^34^. In the proposed method, the channel selection has been carried out using metaheuristic algorithms such as RSA, PSO, SSA, and SO. The features from both classes have been provided to MHAs to obtain the optimal number of channels. The objective of the fitness function is to select an optimal subset of EEG channels that maximizes classification performance while minimizing the number of selected channels. The fitness function is formulated as:

8 $$\begin{aligned} F = \alpha \cdot Acc + \beta \cdot \left( 1 - \frac{N_s}{N_t}\right) \end{aligned}$$

where *Acc* represents the classification accuracy obtained using the selected subset of EEG channels, $N_s$ denotes the number of selected channels, $N_t$ denotes the total number of available channels, $\alpha$ and $\beta$ are weighting coefficients such that $\alpha + \beta = 1$.

This formulation ensures a trade-off between maximizing classification performance and minimizing channel redundancy. The weighting coefficients $\alpha$ and $\beta$ determine the relative importance of classification accuracy and channel reduction in the fitness function. Since the primary objective of this study is to maintain high classification performance while reducing redundant channels, a higher weight is assigned to accuracy. Several combinations of $(\alpha , \beta )$ were evaluated during preliminary experiments, and it was observed that assigning higher importance to accuracy (i.e., $\alpha = 0.7$, $\beta = 0.3$) yields a favorable balance between classification performance and channel reduction. Lower values of $\alpha$ resulted in excessive channel reduction at the cost of reduced classification accuracy, whereas higher values did not significantly improve performance but increased the number of selected channels.

### Classification

ML models can learn the underlying patterns present in the data, make accurate predictions, and enhance the model’s overall performance. In the present study, six classifiers, KNN, SVM, logistic regression, neural network, naive Bayes, and decision tree, are investigated.

**Table 6:** Parameter settings of different algorithms.

Algorithms	Parameter settings
PSO^49^	$\omega _{min}=0.1,\;\omega _{max}=0.9$, and $c_1=c_2=2$
SSA^51^	$c_1,\; c_2\in [1,0]$
RSA^50^	$\theta =0.5,\; \gamma =0.9, \; UB$ and *LB* varied for features
SO^52^	$c_1=0.5, \;c_2=0.05, \;$and $c_3=2.$

**Table 7:** The EEG channels selected using different MHAs.

Algorithm	Channels selected	Channels
SO	29	FP1, F7, FZ, T8, CZ, CP5, CP6, CP1, P3, P4, PZ, PO2, PO1, O2, O1, F6, FT7, FC4, C6, C5, F2, F1, TP8, AFZ, P6, C2, FCZ, OZ, CPZ
PSO	34	FP1, F7, AF2,F4, F3, FC5, FC2, T8, T7, C4, CP5, CP6, CP1, CP2, P4, P7, AF7, AF8, F5, FT7, FC4, C5, CP3, CP4, P5, P6, C1, C2, PO8, POZ, OZ, PZ, P1, CPZ.
RSA	33	F8, AF1, AF2, FZ, F3, FC6, FC2, FC1, T8, C4, CP6, CP1, P4, PZ, PO1, AF7, AF8, F5, F6, FT7, FPZ, FC4, FC3, F2, F1, TP7, P5, C1, PO8, FCZ, OZ, P1, CPZ
SSA	35	F7, AF1, AF2, F3, FC6, T8, T7, C3, CP6, CP1, P3, P4, P7, PO1, O2, O1, AF7, AF8, F5, F6, FT7, FC4, FC3, C5, F2, AFZ, CP4, C1, C2, PO8, FCZ, OZ, PZ, P1, CPZ

**Fig. 5:**
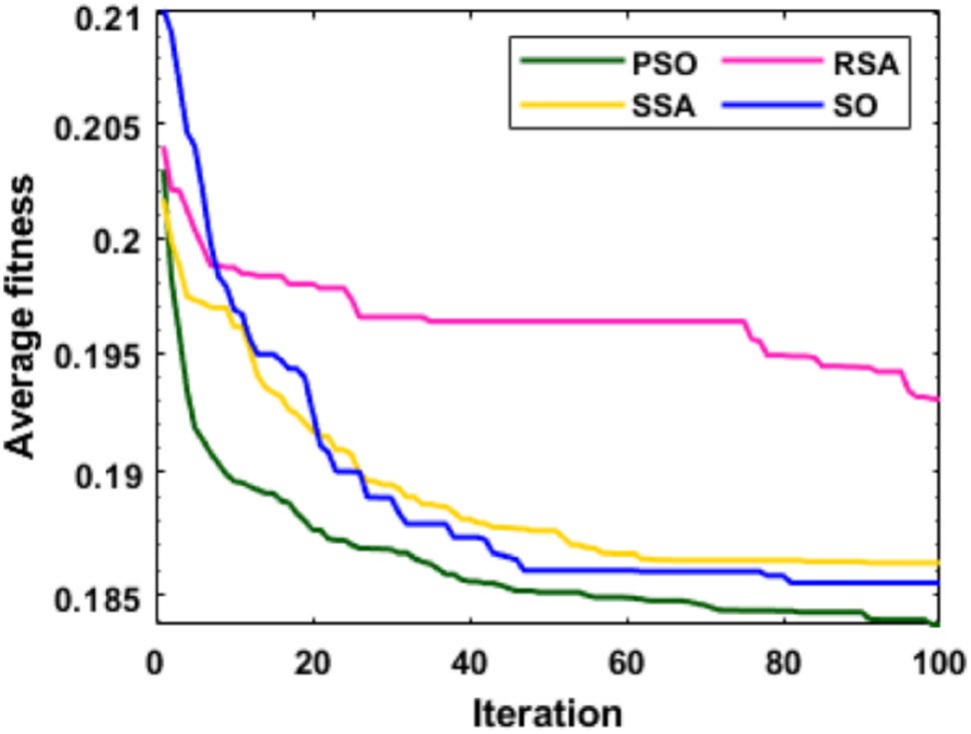
Performance of the PSO, SSA, RSA, and So in terms of average fitness value and number of iterations.

**Table 8:** Fitness values of four channel selection methods.

	PSO	SSA	RSA	SO
Minimum	0.1787	0.1794	0.1737	0.1674
Maximum	0.1897	0.1917	0.2091	0.2031
Mean	0.1838	0.1863	0.1931	0.1855
Std. dev.	0.0037	0.0039	0.0122	0.0129

## Results and discussion

**Table 9:** The performance metrics for the different channel selection algorithms.

	Selected channels	Classifier	Acc. (%)	Sen. (%)	Spe. (%)	Prec. (%)	Mcc. (%)	F1-score (%)
SSA	F7, AF1, AF2, F3, FC6, T8, T7, C3, CP6, CP1, P3, P4, P7, PO1, O2, O1, AF7, AF8, F5, F6, FT7, FC4, FC3,C5, F2, AFZ, CP4, C1, C2, PO8, FCZ,OZ, PZ, P1, CPZ.	KNN	95.90	95.10	97.43	98.63	91.12	96.83
		SVM	94.80	94.38	95.58	97.63	88.70	95.98
		LR	90.93	90.10	92.71	96.34	80.26	93.10
		NN	87.90	90.05	83.90	90.95	73.66	90.50
		NB	75.04	82.39	64.17	77.3	47.46	80.76
		DT	77.20	80.41	70.63	84.82	50.00	82.56
PSO	FP1, F7, AF2,F4, F3, FC5, FC2, T8, 7, C4, CP5, CP6, CP1, CP2, P4, P7, AF7, AF8, F5, FT7, FC4, C5, CP3, CP4, P5, P6, C1, C2, PO8, POZ, OZ, PZ, P1, CPZ.	KNN	95.63	94.58	97.70	98.79	90.58	96.64
		SVM	95.00	94.50	95.62	97.64	88.84	96.02
		LR	91.06	90.10	93.20	96.58	80.57	93.22
		NN	88.20	90.20	85.00	91.38	74.37	90.78
		NB	75.00	82.47	64.00	76.6	47.08	90.78
		DT	76.70	79.75	70.25	84.92	48.60	82.26
RSA	F8, AF1, AF2, FZ, F3, FC6, FC2, FC1, T8, C4, CP6, CP1, P4, PZ, PO1, AF7, AF8, F5, F6, FT7, FPZ, FC4, FC3, F2, F1, TP7, P5, C1, PO8, FCZ, OZ, P1, CPZ	KNN	94.69	93.54	97.05	98.47	88.54	95.93
		SVM	93.65	93.19	94.57	97.13	86.22	95.12
		LR	90.43	89.40	92.50	95.94	79.62	92.55
		NN	87.22	89.42	83.20	90.63	72.25	90.00
		NB	74.57	82.04	63.50	76.76	46.42	79.31
		DT	76.77	78.86	72.00	86.74	49.00	82.61
SO	PFP1, F7, FZ, T8, CZ, CP5, CP6, CP1, P3, P4, PZ, PO2, PO1, O2, O1, F6, FT7, FC4, C6, C5, F2, F1, TP8, AFZ, P6, C2, FCZ, OZ, CPZ.	KNN	93.65	92.27	96.56	98.26	86.30	95.17
		SVM	92.18	91.79	98.97	96.33	83.00	94.00
		LR	88.74	88.03	90.31	95.26	75.42	91.50
		NN	85.85	88.52	81.06	89.35	69.33	88.93
		NB	74.84	82.38	63.81	76.90	47.15	79.55
		DT	77.13	79.14	72.47	87.00	49.50	82.88
All channels		KNN	96.30	94.52	98.30	99.11	90.93	96.83
		SVM	94.80	93.83	96.75	98.30	88.78	95.93
		LR	90.86	89.50	93.88	97.01	80.19	93.22
		NN	88.79	90.99	84.90	91.44	75.73	90.78
		NB	75.20	82.58	64.33	77.35	47.84	90.78
		DT	77.80	79.72	73.38	87.32	50.72	82.61

**Fig. 6:**
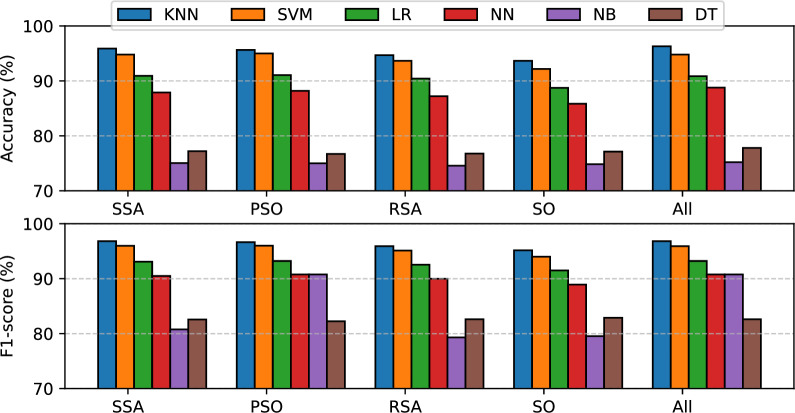
Barplot for the accuracies and F1 Score obtained from different channel selection methods and ML models.

All experiments were conducted on a high performance workstation running Windows 11 (64-bit) with 32GB DDR4 RAM and a multi-core processor Intel Core i7. Initially, all EEG data were pre-processed to remove noise and other distortions. Optimal wavelet is used to get these subbands. The obtained EEG bands from both classes were used as features to obtain the best and most significant EEG channels using several recently demonstrated metaheuristic algorithms. The parameters setting for MHAs is obtained for the maximum performance. The different parameter values for PSO, SSA, SO, and RSA are presented in Table 6. The optimal number of channels was selected using each algorithm and presented in Table 7. The SO selected only 29 channels from 64. The RSA, PSO, and SSA selected 33, 34, and 35 channels, respectively. The minimum, maximum, mean value, and standard deviation fitness values for all four algorithms are reported in Table 8. It is noted from Table 8 that the fitness value for PSO is minimum than SSA, RSA, and SO, i.e., 0.1897, and it is a maximum of 0.2031 for SO. The plot for average fitness value and number of iterations-wise depicted in Fig. 5.

Furthermore, EEG data from significant channels are employed by optimal wavelets to decompose into several subbands. Four distinct features were extracted in order to decrease the redundancy and burden of the ML models. Finally, various ML models were trained and evaluated utilizing the optimum feature set from alcoholic and NC EEG signals. The proposed methods have been tested on the publicly available EEG dataset of alcoholic and normal subjects. The details of this data set are provided in the subsequent subsection II-A.

The accuracy of the performance metrics (Acc.), sensitivity (Sen.), specificity (Spe.), precision (Prec.), Mathew’s correlation coefficient and the F1-score of MHAs have been presented in Table 9. From Table 9, it is observed that the highest accuracy of $95.90\%$ is obtained using KNN for SSA. The other methods, PSO, RSA, and SO, achieved accuracies $95.63\%$, $94.69\%$, and $93.65\%$ using KNN, respectively. However, the SO utilized only 29 channels to obtain $93.65\%$. The highest value of specificity and precision is observed in PSO with KNN. The accuracies for MHAs using KNN, SVM, LR, NN, NB, and DT are plotted in Fig. 6. The SSA utilized 35 channels to achieve maximum performance. Hence, SSA reduced almost 50% of the number of channels. This has reduced the computational cost by $50\%$. The performance of all channels has been investigated to detect alcoholism. This provides an accuracy of $96.30\%$.

Other than KNN, SVM performs better, and it produces accuracies of 95.00%, 94.80%, and 93.65% for PSO, SSA, and RSA, respectively. The lowest accuracy of 74.57% was obtained from NB for RSA. The LR, NN, and DT reached to 90.93%, 87.90%, and 77.20% using an SSA-based feature set.

**Table 10:** Statistical significance analysis using paired t-test for classification accuracy comparison between different channel selection methods with KNN classifier, including number of selected channels and computational cost.

Method comparison	Channels used	Comp. cost	Mean acc. (%)	Std. dev.	p-value	Significant (p < 0.05)
SSA–KNN vs PSO-KNN	35 vs 34	Medium	95.90 vs 95.63	0.32	0.021	Yes
SSA–KNN vs RSA-KNN	35 vs 33	Medium	95.90 vs 94.69	0.45	0.018	Yes
SSA–KNN vs SO-KNN	35 vs 29	Low	95.90 vs 93.65	0.51	0.012	Yes
SSA–KNN vs all channels-KNN	35 vs 64	High	95.90 vs 96.30	0.28	0.094	No

Finally, It is noted that MHAs can reduce the number of channels to obtain the same performance as for all. The SSA with KNN achieved the best performance using 35 channels only. There is a trade-off between accuracy and number of channels. As number of channels are less, the accuracy will be less. However, the proposed model provides only 0.4% less accuracy than all channel feature sets. The proposed method outperforms the previous methods reported in^19,20^ in terms of alcoholism detection. This method can be employed in other neurodegenerative disorders.

### Computational complexity

For all four metaheuristic algorithms (PSO, SSA, RSA, and SO), we used a population size of 30 and 100 iterations. The key parameters were set as follows: SSA awareness probability = 0.7, PSO inertia weight = 0.5, acceleration coefficients c1 = c2 = 1.5, RSA learning rate = 0.5, and SO step size = 0.6. Table 9 reports the fitness values obtained across 20 independent runs, showing that SSA and PSO converge more consistently compared to RSA and SO. The computational cost of each algorithm scales linearly with the number of iterations, population size, and fitness evaluation cost (O(T $\times$ P $\times$ F)). The fitness value itself suggest computational complexity.

The proposed method employs metaheuristic algorithms to identify a reduced subset of EEG channels while maintaining competitive classification performance. For example, the SSA-based channel selection reduces the number of channels from 64 to 35 (approximately a 50% reduction) while still achieving an accuracy of 95.90%. This demonstrates that the proposed method can maintain strong performance while significantly reducing channel redundancy and computational cost this comparison shown in Table 10.

Furthermore, the computational complexity of the proposed approach is reduced, as demonstrated in Table 5. Unlike the standard DWT, which relies on predefined mother wavelets such as Daubechies or Symlets, the proposed approach constructs wavelet filter banks using HBP design based on discrete cosine harmonic functions. This design enables flexible filter bank generation with controllable vanishing moments and improved time–frequency localization, making it more suitable for analyzing non-stationary EEG signals.

### Comparison with existing methods

In the literature summarized in Table 11, various types of mother wavelets and feature extraction techniques have been utilized for alcoholism detection using EEG signals. However, many of these wavelets are predefined and may not optimally represent the characteristics of EEG signals. Therefore, the present study compares the proposed wavelet with existing wavelet-based approaches for the given Table 11 shows that some previously reported methods achieve slightly higher classification accuracies than the present work. However, many of these approaches utilize all available EEG channels or employ computationally intensive signal decomposition techniques. In contrast, the primary objective of the proposed framework is to achieve a balance between classification performance and computational efficiency through optimal EEG channel selection. From the Table 11 it is clear that proposed model uses only 35 channel features to get the 95.90% that significantly reduces the computational complexity.

**Table 11:** Comparison with existing methods.

Ref.	Dataset	Method	Classification model(s)	C*	Acc. (%)
^4^	UCI	Spectral analysis	SVM	64	82.98
^5^	UCI	–	SVM	64	91.70
^6^	UCI	WPT	KNN, GMM, PNN, DT	64	95.80
^7^	UCI	Spectral analysis	SVM, LDA, MLP	64	96.00
^8^	UCI	Time-frequency	NNLS	64	95.83
^9^	UCI	FFT	K-NN	64	95.50
^10^	UCI	EMD rhythms	ELM	64	97.93
^11^	UCI	EMD	Least square SVM	64	97.92
^12^	UCI	Spectral analysis	Ensemble KNN	64	95.10
^13^	UCI	EMD	Ensemble subspace K-NN	64	93.87
^14^	UCI	Wavelet transform	MLP, RBFN, ELM	64	87.60
This work	UCI	OWFB with MHA	KNN	35	95.90

*APEN* approximate entropy, *SAEN* sample entropy, *GMM* Gaussian mixture model, *SVM* support vector machine, *RBFN* radial basis function network, *LS-SVM* least square SVM, *EMD* empirical mode decomposition, *DT-CWT* dual tree complex wavelet transform, *WPT* wavelet packet transform, *Acc* accuracy, *C* number of channels.

Future studies should focus on expanding the dataset using multi-center and longitudinal EEG recordings to improve model generalization across diverse populations and experimental protocols. In addition, transfer learning and pre-trained CNN architectures can be explored to enhance classification accuracy while maintaining computational efficiency for real-time implementation, and applying explainable AI (XAI) methods will provide interpretability to deep models by highlighting critical EEG channels and frequency bands contributing to disease detection.

## Conclusion

Electroencephalography (EEG) offers a means to detect cerebral electrical activity and identify alcoholism. The traditional methods to detect alcoholism are subjective, time-consuming, and prone to error. The proposed research introduces an approach for effective EEG channel selection using MHAs and novel optimal wavelets to decompose the EEG into subbands. From these subbands, four distinct features were extracted. These selected optimal channel features are then employed in the training and testing of ML models. The validation of the proposed method has been carried out using a publicly available alcoholic dataset. The MHAs have reduced the number of channels to $50\%$, achieving the highest performance. The SSA, with only 35 channels, obtained 95.90% accuracy. Remarkably, when paired with a KNN, the proposed approach achieves an impressive 95.90% accuracy rate using the SO from only twenty-nine channels. The MHAs with optimal wavelets demonstrate their efficacy in achieving high accuracy in detecting alcoholic states using a minimal set of features. It enhances the performance of the ML models and reduces the computational time compared to existing alcoholism detection methods. Furthermore, the proposed optimal wavelets can be employed to broader application in the treatment of other neurodegenerative disorders where EEG decomposition is required.

## Data Availability

The publicly available EEG datasets of alcoholic and normal controlled subjects used in the study have been taken from KDD UCI, University of California (https://kdd.ics.uci.edu/databases/eeg/eeg.data.html)
